# Association between weight perception and socioeconomic status among adults in the Seychelles

**DOI:** 10.1186/1471-2458-10-467

**Published:** 2010-08-09

**Authors:** Heba Alwan, Bharathi Viswanathan, Julita Williams, Fred Paccaud, Pascal Bovet

**Affiliations:** 1 Institute of Social and Preventive Medicine (IUMSP), University Hospital Centre and University of Lausanne, Rue du Bugnon 17, 1005 Lausanne, Switzerland; 2 Ministry of Health and Social Development, Victoria, Republic of Seychelles

## Abstract

**Background:**

Few studies have examined the association between weight perception and socioeconomic status (SES) in sub-Saharan Africa, and none made this association based on education, occupation and income simultaneously.

**Methods:**

Based on a population-based survey (n = 1255) in the Seychelles, weight and height were measured and self-perception of one's own body weight, education, occupation, and income were assessed by a questionnaire. Individuals were considered to have appropriate weight perception when their self-perceived weight matched their actual body weight.

**Results:**

The prevalence of overweight and obesity was 35% and 28%, respectively. Multivariate analysis among overweight/obese persons showed that appropriate weight perception was directly associated with actual weight, education, occupation and income, and that it was more frequent among women than among men. In a model using all three SES indicators together, only education (OR = 2.5; 95% CI: 1.3-4.8) and occupation (OR = 2.3; 95% CI: 1.2-4.5) were independently associated with appropriate perception of being overweight. The OR reached 6.9 [95% CI: 3.4-14.1] when comparing the highest vs. lowest categories of SES based on a score including all SES indicators and 6.1 [95% CI: 3.0-12.1] for a score based on education and occupation.

**Conclusions:**

Appropriately perceiving one's weight as too high was associated with different SES indicators, female sex and being actually overweight. These findings suggest means and targets for clinical and population-based interventions for weight control. Further studies should examine whether these differences in weight perception underlie differences in cognitive skills, healthy weight norms, or body size ideals.

## Background

Weight perception is known to be associated with a number of factors including sex [1-3], race [1-7], actual weight status [5,6,8] and socioeconomic status (SES) [1-3,5,9-11]. While the relationship between weight perception and SES has been assessed in several Western countries [1-3,5,9], few such studies have been conducted in sub-Saharan Africa [10,12-14]. The existing literature in both Western countries and Africa indicates that appropriate perception of one's own weight is more frequent in high than low SES individuals [1-3,5,9,14].

An appropriate perception of one's own weight is conducive to improved weight control behavior [15,16]. Better knowledge on the determinants of perception of one's own weight may thus be important in weight control strategies. In addition to appropriate self-perceived weight, body size dissatisfaction [15,16] and ideal body weight [15] are other potential factors that may play a role in determining an individual's weight-control behavior. The situation in the Seychelles, a middle-income country, provides an interesting case study as the association between obesity and SES is direct in men, but inverse in women [17]. Therefore, gender-related differences in weight across SES categories may contribute to the differential obesity-SES relationship in men and women in the Seychelles. In contrast, obesity tends to be more prevalent among both men and women of low SES in developed countries [18].

Few studies have compared the association between weight perception and different SES indicators. Previous reports indicate that the association between SES and weight perception [1,5] and body dissatisfaction [19] was stronger based on education than on income or occupation, where education was positively associated with a higher self-perceived weight status and body weight dissatisfaction. We are not aware of any study that has compared the association between weight perception and education, occupation and income in sub-Saharan Africa.

In this study, we examined the association between one's own weight perception and SES indicators in individuals randomly selected from the population in a rapidly developing country in sub-Saharan Africa, and whether this association differed based on education, occupation, and income.

## Methods

The Republic of Seychelles is a group of islands located approximately 1800 km east of Kenya. The national gross domestic product per capita increased, in real terms, from US$ 2927 in 1980 to US$ 5239 in 2004. The Seychelles is considered as an upper middle-income country. The majority of the population is of African descent and 90% of the total population lives on the largest island.

The data in this paper come from the Seychelles Heart Study III, a population-based survey conducted in 2004 under the auspices of the Ministry of Health of the Republic of Seychelles. Detailed methods and results of the survey have been described previously [20], including the population distribution of body weight and main cardiovascular risk factors [21] and the association of body weight with SES [17]. Briefly, eligible participants aged 25-64 years were selected from computerized data of a national population census in 2002 thereafter updated by civil status authorities. The survey was attended by 1255 individuals, corresponding to a participation rate of 80% [21]. The survey was approved by the Ministry of Health after technical and ethical reviews. Participants were free to participate and gave written informed consent.

A structured questionnaire was administered by experienced nurses to all participants through a face-to-face interview. Weight perception was assessed using the question: "Do you think your weight is: largely too high, a bit too high, good, or too low?"

Weight and height were measured using precision electronic scales (Seca™, Hamburg, Germany) and fixed stadiometers (Seca™). Body mass index (BMI; kg m^-2^) categories were defined as follows: underweight: <18.5, normal weight: 18.5-24.9, overweight: 25-29.9, and obesity: ≥30 [22].

In this paper, three SES indicators were categorized into three ordered categories: 'low', 'intermediate', and 'high' categories, on the basis that there were naturally well defined categories and/or the categories divided the population into fairly even numbers. For education, the three categories were attendance of primary school, secondary school, and post secondary education. For professional occupation, the low category included laborers (i.e. manual occupation with no formal training), the high category included professionals and non-manual occupations with formal training (e.g. teachers, nurses, etc), while the intermediate category included all other professional occupations. Occupation referred to the current occupation or to the most recently held occupation if the individual was currently not working for a wage. Of note, more than 80% of men and women aged 25-64 reported to currently have a job. Monthly income related to the reported occupation was trichotomized into three categories including fairly even numbers of persons (i.e. <2000 rupees, 2000-5000 rupees, and >5000 rupees; 1 US$ ~8 rupees in 2004).

Weight perception was stratified by sex, actual weight status, and SES. A proportional Venn diagram was used to display agreement between the upper categories of the three SES indicators [23]. The association between weight perception and SES was assessed using logistic regression adjusted for age, sex, and actual weight status. Cusick's trend test was used to test for trends in weight perception across the SES categories. Two summary SES scores were calculated by adding answers of the SES indicators (coding 0, 1, or 2 for 'low', 'intermediate', and 'high' categories, respectively). The first score took into account education and occupation, and therefore ranged from 0 (low level for education and occupation) to 4 (high level for education and occupation). The second score took into account all three SES indicators (education, occupation, and income), and ranged from 0 (low level for all three indicators) to 6 (high level for all three indicators). The association between weight perception and the SES score was assessed using logistic regression adjusted for age, sex, and actual weight status. All analyses in this paper were restricted to the individuals (n = 1239) who had full data for all variables relevant to this paper. Weight perception was not assessed for underweight individuals (i.e. BMI <18.5). Since results for the association analyses were virtually unchanged whether data were weighted or not to the age structure of the population, data in this paper are not weighted for age, unless specified otherwise (i.e. overall prevalence of BMI categories) [24]. Statistical analyses were performed using Stata 9.2 and p values <0.05 were considered significant.

## Results

Overall, 3.5% of the participants were underweight, 33.4% were normal weight, 35.3% were overweight, and 27.9% were obese. The age-adjusted prevalence of these categories was 3.9%, 36.0%, 35.0% and 25.1%, respectively. Among normal weight participants, 6.5% (7.6% of males and 5.1% of females) inappropriately perceived their weight as being too low. Among persons with excess weight, 54% of overweight participants (63.5% of males and 45.1% of females) and 18.8% of obese participants (23.6% of males and 17.2% of females) failed to perceive their weight as being too high. Moreover, it should be noted that even a belief that one's weight is "a bit to high" (vs. 'largely too high") may actually be somehow inappropriate among obese persons.

**Figure 1:**
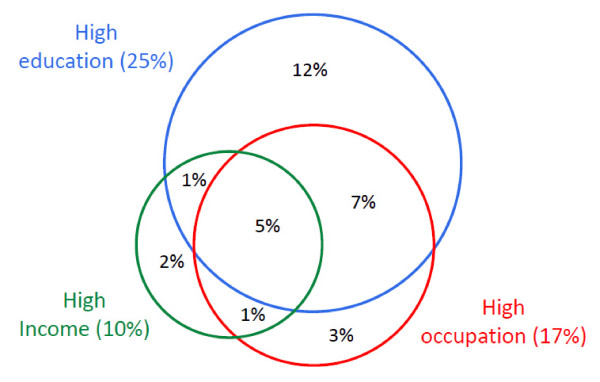
**Level of agreement between the 'high' categories of the three socioeconomic status (SES) indicators**. * 32% of all participants belonged to the 'high' SES group based on any of the three SES indicators. ** The values in parentheses represent the proportion of individuals falling in the highest category of each SES indicator.

Figure 1 shows poor agreement between the upper categories of three SES indicators among all study participants. The proportions of persons in the highest SES category were 25% based on education, 17% based on occupation, 10% based on income, 32% based on any of the three indicators and only 5% based on all three indicators. This latter proportion (high SES based on all three indicators) represented only 16% of those identified as high SES based on any single SES indicator.

**Figure 2:**
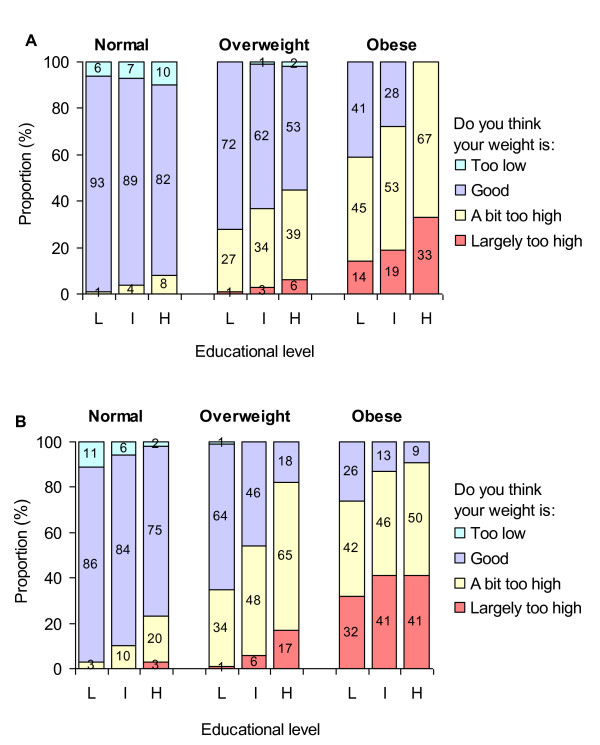
**Self-perceived body weight according to sex, actual weight status, and education**. L: low, I: intermediate, H: high. Panel A: men, Panel B: women.

Figure 2 shows the distribution of categories of body image perception by sex, actual BMI, and education. Perception of one's weight as too high was more frequent in women than men (irrespective of BMI categories) and in obese than overweight individuals (irrespective of sex). Among overweight/obese participants, perception of having a weight that is too high was more frequent among persons of high vs. low education, irrespective of sex.

**Figure 3:**
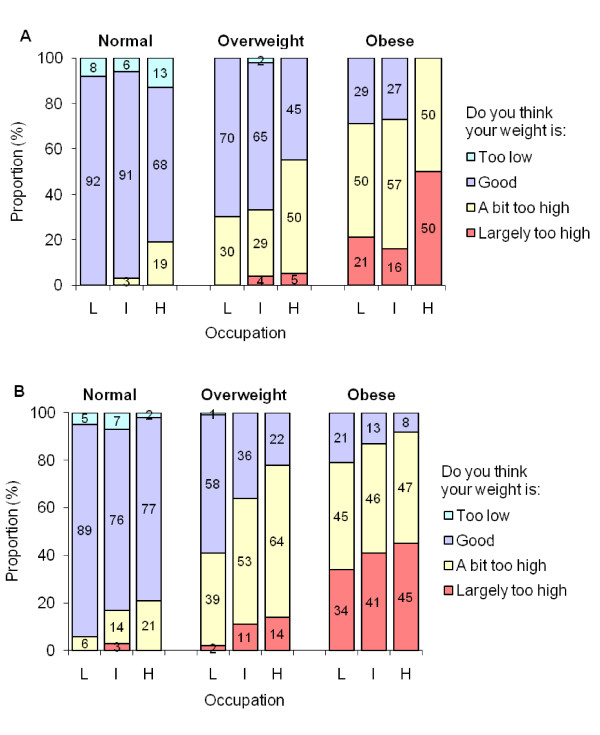
**Self-perceived body weight according to sex, actual weight status, and occupation**. L: low, I: intermediate, H: high. Panel A: men, Panel B: women.

**Figure 4:**
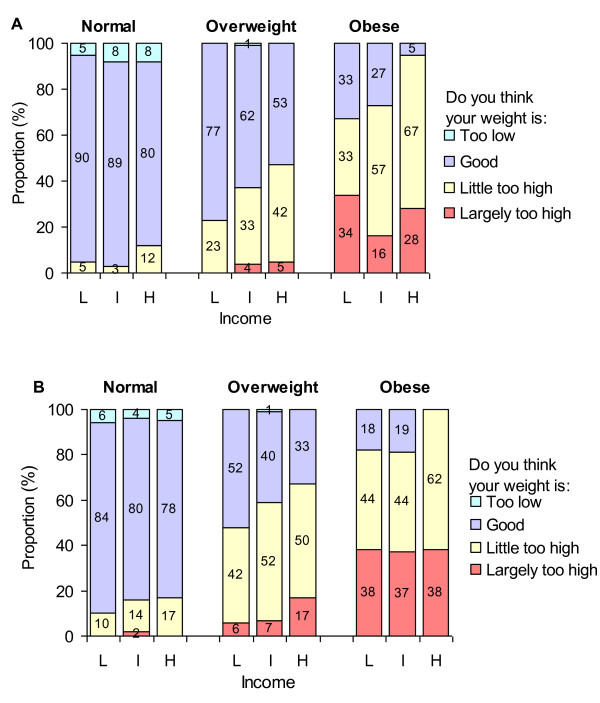
**Self-perceived body weight according to sex, actual weight status, and income**. L: low, I: intermediate, H: high. Panel A: men, Panel B: women.

Similar associations were found using occupation and income (Figures 3 and 4): perceiving one's weight as too high was reported more often by women than men, by obese than overweight participants, and by individuals of high than low SES. Table 1 shows a statistically significant trend in appropriately perceiving one's weight as too high across increasing SES categories. This trend was observed for both overweight and obese individuals among men and women (p ≤ 0.05 in most cases) for education and occupation but was less clear when income was used as an SES indicator. Of note, while perception of having a weight that is too high was positively associated with SES in both overweight and obese individuals, obese participants more frequently reported their weight as too high at each SES level compared to their overweight counterparts.

**Table 1:** Percentage of overweight or obese individuals who perceived their weight as too high according to sex, actual weight status, and socioeconomic status (SES).

		Education			Occupation			Income		
				
Sex	SES	N	%	p	N	%	p	N	%	p
*Overweight*										
M	Low	60	28.3	0.062	44	29.5	0.016	31	22.6	0.038
	Intermediate	98	36.7		125	32.8		144	36.8	
	High	53	45.3		42	54.8		36	47.2	
										
F	Low	68	35.3	0.000	110	40.9	0.000	93	48.4	0.087
	Intermediate	107	54.2		80	63.8		121	58.7	
	High	51	82.4		36	77.8		12	66.7	
										
*Obese*										
M	Low	22	59.1	0.001	14	71.4	0.105	15	66.7	0.055
	Intermediate	43	72.1		63	73.0		56	73.2	
	High	24	100.0		12	100.0		18	94.4	
										
F	Low	98	73.5	0.003	152	79.0	0.039	119	82.4	0.433
	Intermediate	112	87.5		68	86.8		124	81.5	
	High	46	91.3		36	91.7		13	100.0	
										
Overweight or obese										
M	Low	82	36.6	0.001	58	39.7	0.009	46	37.0	0.009
	Intermediate	141	47.5		188	46.3		200	47.0	
	High	77	62.3		54	64.8		54	63.0	
										
F	Low	166	57.8	0.000	262	63.0	0.000	212	67.5	0.157
	Intermediate	219	71.2		148	74.3		245	70.2	
	High	97	86.6		72	84.7		25	84.0	

Table 2 shows the odds ratios (OR) for perceiving one's weight as too high according to SES among overweight/obese individuals. All ORs are adjusted for sex, age, and actual weight status. Appropriate perception of a high self-perceived weight was associated with sex (OR between 2.1-2.4 for women vs. men, depending on which SES indicator was used) and with actual weight status (OR between 4.9-5.4 for obese vs. overweight individuals). The ORs for the three SES indicators were not largely different depending on which SES indicator was used. However, the association tended to be largest for high vs. low education categories (OR = 4.3; 95% CI: 2.5-7.3), intermediate for high vs. low occupation categories (OR = 4.0; 95% CI: 2.4-6.8) and weakest for high vs. low income categories (OR = 2.7; 95% CI: 1.5-5.1). These results suggest that any of the three SES indicators may be similarly useful to predict appropriate weight perception when used in isolation.

**Table 2:** Association between high self-perceived weight and socioeconomic status (SES) among overweight or obese persons

		Education		Occupation		Income		All 3 SES indicators	
					
	N	OR	P	OR	P	OR	P	OR	P
Sex									
Men	300	1		1		1		1	
Women	482	2.13	0.000	2.39	0.000	2.26	0.000	2.36	0.000
Age									
25-44	323	1		1		1		1	
45-64	459	0.85	0.420	0.55	0.001	0.52	0.000	0.72	0.110
Body mass index									
Overweight	437	1		1		1		1	
Obese	345	5.20	0.000	5.38	0.000	4.93	0.000	5.39	0.000
Education									
Primary	248	1		-	-	-	-	1	
Secondary	360	1.88	0.002	-	-	-	-	1.54	0.055
Postsecondary	174	4.26	0.000	-	-	-	-	2.46	0.009
Occupation									
Laborer	320	-	-	1		-	-	1	
Intermediate	336	-	-	1.60	0.017	-	-	1.35	0.157
Professional	126	-	-	4.01	0.000	-	-	2.27	0.018
Income									
Low	258	-	-	-	-	1		1	
Intermediate	445	-	-	-	-	1.18	0.376	0.88	0.520
High	79	-	-	-	-	2.73	0.002	1.21	0.598

* Each model (column) is adjusted for all variables displayed in that column.

In multivariate analysis adjusting for age, sex, and actual weight status and the three SES indicators, only education (OR = 2.5; 95% CI: 1.3-4.8) and occupation (OR = 2.3; 95% CI: 1.2-4.5) remained significantly associated with perception of having a weight that is too high. Strong associations were also found for sex (OR 2.4 for women vs. men) and actual weight status (5.4 for obese vs. overweight). These results suggest independent effects for education and occupation, as well as for female sex and for being obese.

**Figure 5:**
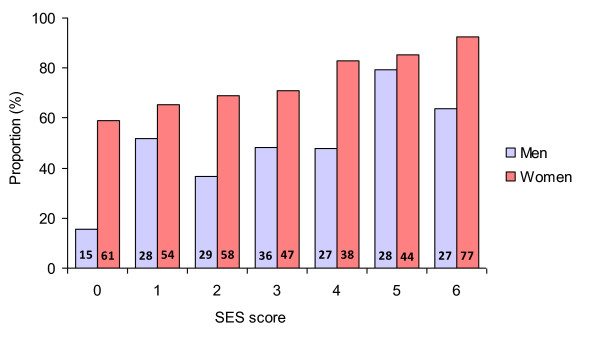
**Percentage of overweight and obese individuals who perceived their weight as too high according to a score based on all three socioeconomic status (SES) indicators and sex**. * Figures shown within bars are the proportions of obese persons as a percent of all persons with overweight or obesity within each sex and SES strata.

Figure 5 shows the proportions of overweight/obese men and women appropriately reporting that their weight is too high according to the SES score based on all three SES indicators. Perception of having a weight that is too high increased gradually along the SES score. Consistent with the previous results, women more frequently perceived their weight as too high across all categories of the SES score compared to men.

Table 3 displays the multivariate association between perceiving one's weight as too high, among overweight/obese persons, and the SES scores based on either education and occupation (column 1) or all three SES indicators (column 2). Categories 5 and 6 of the score based on the three SES indicators were combined together in order to maximize sample size as there were only 70 and 35 persons in the 5^th ^and 6^th ^categories, respectively. The odds of an overweight/obese individual to perceive his/her weight as too high gradually increased along the two SES scores and was 6.1 (95% CI: 3.0-12.1) (column 1) and 6.9 (95% CI: 3.4-14.1) (column 2) comparing participants with a high vs. low SES score. Again, perception of a high weight was also independently associated with being a woman (vs. men) and being obese (vs. overweight). Consistent with results in Table 1 factoring all three SES indicators (showing redundancy of income once education and occupation are factored), the OR between the lowest and highest categories of a score including only education and occupation (i.e. low education and occupation vs. high education and occupation) yielded an OR that was only slightly lower than the OR between extreme categories of the score including the three SES indicators.

**Table 3:** Association between high self-perceived weight and two socioeconomic status (SES) scores among overweight or obese persons

	Score based on education and occupation (out of 4)			Score based on education, occupation, and income (out of 6)		
		
Variable	N	OR	P	N	OR	P
Sex						
Men	300	1		300	1	
Women	482	2.43	0.000	482	2.43	0.000
Age						
25-44	323	1		323	1	
45-64	459	0.75	0.121	459	0.67	0.030
Body mass index						
Overweight	437	1		437	1	
Obese	345	5.44	0.000	345	5.45	0.000
SES score						
0	176	1		118	1	
1	206	1.65	0.043	141	1.74	0.054
2	200	2.16	0.003	156	1.50	0.172
3	110	3.75	0.000	162	2.02	0.021
4	90	6.06	0.000	100	3.08	0.001
5 or 6	-	-	-	105	6.91	0.000

## Discussion

We found that reporting a high self-perceived weight, among persons who actually were overweight or obese, was more frequent in women than men, in obese than overweight persons, and in persons of high vs. low SES. The association between appropriate perception of one's own weight and SES was fairly similar for all three SES indicators (i.e. education, occupation or income) when these SES indicators were considered in isolation, but the association was no longer significant for income when all three indicators were considered together. This suggests that any SES indicator is a useful marker when information is not available on the other SES indicators but that income does not add substantial information when a person's education and occupational status are known. Correspondingly, the OR for the association between weight perception and SES ranged between 2.7 and 4.3 when education, income or occupation were considered in isolation and reached 6.1 based on a score combining education and occupation and 6.9 based on a score combining these three SES indicators.

Among individuals with a normal BMI, the majority had an appropriate weight perception, i.e. they perceived their weight as being 'good'. It is worth noting however, that high SES individuals more frequently overestimated their weight (i.e. reported a high self-perceived weight when their actual weight was normal). This is consistent with previous reports showing that high SES persons, particularly women, tend to be less satisfied with their weight [19], and are more likely to overestimate their weight and/or underestimate what a normal weight should be [1,3,5].

Obese participants perceived their weight as too high more often than overweight participants (81% vs. 46%, respectively). These results are in line with previous studies showing that obese individuals are less likely to misclassify their weight status as compared to overweight individuals [5,6,8]. Consistent with previous reports [2,6,8], women were more likely than men to appropriately report a high self-perceived weight, regardless of SES. These associations of appropriate weight perception with sex (women vs. men) and weight status (obese vs. overweight) can also underlie different body ideals in women than men (as women have smaller body ideals than men) [1,25] and larger readiness to acknowledge excess weight among obese than overweight persons [5,6].

We found that overweight/obese individuals of high SES were more likely to have an appropriate perception of their excess weight. The association between appropriate self-perception of weight and high SES has been previously documented [1,3,5,6,9], and may be attributed to differences in defining 'normal' or 'ideal' body weight across SES groups [11,19,26]. Moreover, individuals with high SES tend to have greater access to health information that promotes healthy lifestyles, thus rendering these individuals more weight-conscious [3] and more prone to recognize excess weight along the standard overweight and obesity categories. This suggests that interventions that aim to address individuals' weight perceptions can be specifically targeted at low SES groups. Interestingly, both men and women had a more appropriate perception of their weight if they were of high than low SES. The fact that the prevalence of obesity is higher in men of high than low SES suggests, however, that factors other than weight perception underlie the direct obesity-SES relationship among men in the Seychelles. Inversely, the prevalence of obesity is higher among women of low than high SES in the Seychelles, which is consistent with the social gradient in weight perception. More generally, the social pattern in the prevalence of obesity in men and women may also be consistent with a trend for women to increasingly value a lean weight in developing countries, while men may value a heavy weight as a sign of physical dominance and prowess [18]. These observations emphasize the potential role of beliefs and values related to one's weight when assessing social trends in obesity in populations [9,18].

While previous reports have shown that the SES-obesity relationship is more apparent when using education and occupation as SES indicators [18], there remains some controversy as to which of the three SES indicators is the most strongly associated with appropriate weight perception. One previous study showed that education was a stronger contributor to body dissatisfaction than occupation [19] (of note, body dissatisfaction is not fully equivalent with appropriate body perception: a person (e.g. a man) can be aware of being too heavy from a health perspective but still be satisfied with a heavy weight). In our study, we found that any of the three considered SES indicators, i.e. education, occupation, and income, were fairly similarly associated with appropriate weight perception. This means that for practical purposes, any of the SES indicators can be useful to anticipate appropriate perception of a person's own weight, if only one such indicator is available. However, analysis including all three indicators together showed that only education and occupation were independently associated with appropriate weight perception, suggesting that information on income is not necessary if education and occupation are known. This message is also conveyed by the finding that the association was much stronger based on a score combining all three single SES components (OR = 6.9), or a score based on education and occupation (OR = 6.1), than based on any single SES variable (OR between 2.7 and 4.3). Of note, the three SES indicators identified largely different persons and only 16% of individuals placed in the 'high' SES category based on any of the three SES indicators had a high level for all three indicators. It can be observed that quintiles or sixtiles of the two considered overall SES scores imply smaller numbers in the outer score categories as compared to the numbers of persons in the outer categories of the trichotomized scores based on one SES component (education, occupation, or income). The use of more stringent categories in the case of the overall scores vs. the one-component scores may explain the higher ORs in the former than the latter scores. Since our data do not allow us to generate scores of education and occupation that are composed of more than 3 categories, we cannot simulate the ORs that would arise from having narrower categories for these single component scores. Overall, our figures suggest that the one-component scores perform well (particularly the scores based on education and occupation) but factoring knowledge from all three indicators may possibly slightly improve the prediction.

The finding that weight perception was more strongly associated with education and occupation vs. income may reflect differences in health literacy across educational groups [27]. Individuals with a high education may be more able to interpret and use information related to 'healthy' weight and weight control measures [28] compared to individuals with a lower education.

Our findings provide further evidence on phenomenological mechanisms that can fuel the obesity epidemic in the population in this region, and clues to guide interventions to prevent and control overweight and obesity. At a clinical level, our data suggest that health professionals should systematically clarify their patients' beliefs related to their own weight and address the identified related misbelieves. At a population level, our findings suggest that it is important to gather information on weight perception in populations according to various dimensions (gender, SES; etc) in order to guide information campaigns and other culturally sensitive interventions related to a healthy weight.

Strengths of this study include the population-based design and the availability of three SES indicators reflecting three main domains, education, occupation and income. Moreover, weight and height were actually measured, in contrast to a number of similar studies that have relied on self-reported values. On the other hand, the cross-sectional design of this study limits inference on the direction of the associations (i.e. whether low SES leads to poor weight perception or whether poor weight perception -possibly a marker of other poor cognitive skills- leads to poor SES outcomes). Also, as we did not have data on ideal body size, health awareness or cognitive skills (e.g. abstraction skills), we could not disentangle whether differences in appropriate weight perception corresponded to differences in cognitive skills, healthy weight awareness, or body size ideals.

## Conclusions

Appropriately perceiving one's weight as too high was strongly associated with different SES indicators, female sex and being actually overweight. Given the association between appropriate perception of one's own weight and adequate weight-related behavior [15,16], our results suggest means and targets for clinical and population-based weight control programs. Further studies should examine whether these differences in weight perception underlie differences in cognitive skills, healthy weight norms, or body size ideals.

## Competing interests

The authors declare that they have no competing interests.

## Authors' contributions

HA led the analysis of data and write up of the manuscript; BV and JW conducted the interviews and reviewed the manuscript; FP participated in the study design and reviewed the manuscript; PB was the PI of the survey and actively participated in data analyses and in the write up of the manuscript. All authors have read and approved the manuscript.

## Pre-publication history

The pre-publication history for this paper can be accessed here:

http://www.biomedcentral.com/1471-2458/10/467/prepub
